# Understanding barriers: patient experience with immune-mediated rheumatic diseases in Brazilian public health

**DOI:** 10.1186/s42358-026-00563-x

**Published:** 2026-08-19

**Authors:** Helena Tupinambá, Leticia Rocha Pereira, Carla Gottgtroy, Blanca Elena Rios Gomes Bica, Ingrid Bandeira Moss, Gabriela Sadigurschi, Ewerton Alves Portela dos Santos, Janaina Reis Xavier, Maria Cristina Caetano Kuschnir, Vitor Cardoso Doria da Gama, Thalita da Matta de Castro, Maria de Lourdes de Sousa Maia

**Affiliations:** 1 Rheumatology Division, Internal Medicine Department, Pedro Ernesto University Hospital, State University of Rio de Janeiro (HUPE/UERJ), Boulevard Vinte e Oito de Setembro 77 , Rio de Janeiro, RJ 20551-030 Brazil; 2 Department of Medical Affairs, Clinical Studies, and Post-Registration Surveillance (DEAME), Bio-Manguinhos/Oswaldo Cruz Foundation (FIOCRUZ), Av. Brasil 4365, Rio de Janeiro, RJ 21040-900 Brazil; 3 Rheumatology Division, Federal University of Rio de Janeiro (UFRJ), Clementino Fraga Filho University Hospital, Prof. Rodolpho Paulo Rocco Street n. 255, Rio de Janeiro, RJ 21941-590 Brazil; 4 Faculty of Medical Sciences, State University of Rio de Janeiro (FCM/UERJ), v. Prof. Manoel de Abreu, 444 – Maracanã, Rio de Janeiro, RJ 20550-170 Brazil

**Keywords:** Rheumatology, Rheumatic diseases, Diagnosis, Disease Management, Therapeutics, Barriers

## Abstract

**Background:**

Immune-mediated rheumatic diseases (IMRDs) encompass a wide range of rheumatological conditions that have a substantial impact on morbidity and mortality globally. Due to the diversified nature of IMRD symptoms, timely recognition of these conditions in primary care settings can be challenging. The duration between symptom onset and treatment initiation is a key factor influencing prognosis of IMRD. Therefore, this study aims to evaluate the medical journey of individuals with IMRD from symptom onset to treatment.

**Methods:**

Cross-sectional observational study based on the analysis of questionnaire responses from 1,327 patients with IMRDs at two reference centers for rheumatic disease treatment in Rio de Janeiro. Quantitative variables were compared using the Mann-Whitney or Kruskal-Wallis test, while categorical and nominal variables were analyzed using McNemar’s test.

**Results:**

The median time until the first rheumatologist consultation after symptom onset was 7 months (0,5-216 months) and the median time to obtain a definitive diagnosis of IMRD was 12 months (0,5-216 months). This period was shorter for individuals with systemic lupus erythematosus (SLE), with a median of 8 months (0.5–216 months), and longer for those with psoriatic arthritis (PsA), with a median of 33 months (2-195 months). Additionally, the median time from symptom onset to specific treatment initiation was also 12 months. It was shown that consulting two or more non-rheumatologists doctors before seeing a rheumatologist significantly delayed the IMRD diagnosis (p-value < 0.001). The diagnosis of IMRD had a negative impact on emotional well-being and occupational capacity on 85.9% (*n* = 1,139) of participants’ and 68.1% (*n* = 903) indicated that their work was somehow affected in a negative way. Throughout the diagnostic journey, negative feelings predominated in the period in which the diagnosis was not defined, but there was a notable shift after the confirmation of the diagnosis with an increase in positive emotions such as happiness, comfort, and well-being.

**Conclusions:**

This study provides valuable information on the challenges faced in the medical journey of patients with IMRDs, from delays in diagnosis to barriers in accessing treatment and the impact on well-being, highlighting the need for multifaceted interventions to improve the management of these complex conditions.

**Supplementary Information:**

The online version contains supplementary material available at https://doi.org/10.1186/s42358-026-00563-x.

## Background

Immune-mediated rheumatic diseases (IMRDs) represent a heterogeneous group of rheumatological conditions that significantly impact morbidity and mortality worldwide. The Global Burden of Disease Study showed that in 2020, Rheumatoid arthritis (RA) accounted for 3,060,000 disability-adjusted life years (DALYs), with an age-standardized rate of 36.4 DALYs per 100,000 people [1]. Another study carried out by the Lithuanian National Registry found that patients with inflammatory rheumatic diseases have an age- and sex-adjusted standardized mortality ratio (SMR) of 1.32, indicating a 32% higher mortality risk compared to the general population [2].

The underlying mechanisms of IMRDs are not yet fully understood [3]. However, studies have shown that the pathogenesis of these disorders is multifactorial [4–7]. There are more than 30 recognized IMRDs, with rheumatoid arthritis (RA), spondyloarthritis (SpA), systemic lupus erythematosus (SLE), vasculitis, autoimmune myopathies, Sjögren’s syndrome (SS), and systemic sclerosis (SSc) among the most prevalent examples [8]. The pathological process of IMRD depends on the type of disorder, which can affect different target cells and/or human tissues. Given this variability of symptoms, the rapid recognition of different pathologies in primary care settings becomes difficult for non-rheumatologist healthcare providers, which often results in delayed diagnosis [9]. In the context of IMRDs, the time elapsed from symptom onset to the initiation of treatment is a critical determinant of prognosis [10].

In Brazil, rheumatic diseases pose a significant burden on public health and the economy. The REAL study (2018), prospective research on rheumatoid arthritis patients conducted by Pinheiro et al. in Brazil, highlighted significant delays in diagnosis and initiation of disease-modifying antirheumatic drugs (DMARDs), which could explain the high prevalence of moderate or high disease activity and erosive disease. The study also emphasized that early diagnosis and treatment initiation in RA patients are heavily reliant on timely access to rheumatologists [11]. In addition to economic concerns, the social impact of IMRDs is profound. The literature reports a high prevalence of psycho-emotional disorders, such as depression, anxiety, and stress, among IMRD patients, which can exacerbate disease progression and hinder treatment adherence [12].

The formal gateway to the health system should be primary care, where patients are referred to specialists after first evaluation. Even in areas covered by primary care, there is low clinical resolution and difficulty in implementing care coordination [13]. This process often results in prolonged delays in referrals to rheumatologists, missing the critical window for early intervention, thereby adversely affecting long-term prognoses and disease outcomes [14].

### Objectives

The primary objective of this study was to map the patient journey of individuals with IMRDs within the public health system of Rio de Janeiro. Specifically, we aimed to: (1) quantify the time intervals from symptom onset to first medical evaluation, specialist referral, and definitive diagnosis; (2) identify structural barriers contributing to diagnostic delays; and (3) assess the subsequent time to treatment initiation following diagnosis.

## Methods

This observational, cross-sectional survey with retrospective data collection, conducted from May 2022 to September 2023 and sponsored by Bio-Manguinhos/Fiocruz, is based on the analysis of questionnaire responses from patients with IMRDs at two reference centers for rheumatic disease treatment in Rio de Janeiro: Pedro Ernesto University Hospital (*Hospital Universitário Pedro Ernesto* or HUPE) and Piquet Carneiro Polyclinic (*Polyclinic Piquet Carneiro* or PPC) from the Rio de Janeiro State University - and Clementino Fraga Filho University Hospital (*Hospital Universitário Clementino Fraga Filho* or HUCFF) from the Rio de Janeiro Federal University. Patients were recruited at the rheumatology outpatient clinics of both units, at PPC’s infusion center and at the diagnostic center of the HUCFF. Potential research participants were invited to join the study while awaiting their consultation, treatment, or complementary examination. Those who consented to participate signed an informed consent form.

The inclusion criteria for the study involved patients with IMRDs such as RA, psoriatic arthritis (PsA), ankylosing spondylitis (AS) and other SpA, SLE, SSc, APS, primary SS, vasculitis, and autoimmune myopathies. The diagnosis of these conditions had to be reported by the patient and confirmed through medical records at participating institutions. Additionally, only patients over 18 years of age, of both sexes, and residing in the state of Rio de Janeiro were included. Patients with other non-IMRDs or without a confirmed diagnosis of an IMRD were excluded from the study.

The research instrument used to obtain data was a comprehensive questionnaire developed based on a review of similar tools published in the literature and the expertise of rheumatologists and pharmacists involved in its creation. The final instrument covers aspects such as sociodemographic information, questions addressing access to comprehensive treatment, and follow-up after diagnosis. Prior to full data collection, a pilot study was conducted with the first 30 patients at the coordinating center to assess comprehension and identify missing information, resulting in minor adjustments to the instrument. To ensure data accuracy and patient understanding, the final electronic questionnaire (Bio-Manguinhos’ Bioform) was administered through interviews conducted by trained researchers, including physicians and nurses, who were available to clarify doubts (Supplementary Material 1).

Frequencies and percentages were calculated for categorical variables, while mean, median, standard deviation, and range were calculated for continuous variables. Data analysis was stratified by diagnosis to investigate the existence of disease-specific patterns in patient medical journeys. Comparisons of quantitative variables were performed using the Mann‒Whitney or Kruskal‒Wallis test, and comparisons of qualitative variables were conducted using McNemar’s test, with a significance level set at *p* < 0.05. The software employed for analysis was IBM SPSS Statistics 20.0 (IBM Corp. Released, 2011).

## Results

A total of 1,330 patients were invited to participate in the study, out of which 1,327 were eligible to respond to the research questionnaire. Three individuals were deemed ineligible due to the lack of a confirmed diagnosis. Among the 1,327 participants analyzed, 1,142 were female (86.1%) and 185 were male (13.9%), with a median age of 53 years (ranging from 18 to 93 years). Most participants reported a monthly household income of up to two minimum wages, with smaller proportions earning higher income levels. The study identified RA (35%, n = 464) and SLE (34%, n = 451) as the most prevalent diagnoses among participants with IMRDs. In terms of overlapping diagnoses, 57 patients had more than one condition, with the most common combination being SLE + SS (33%, n = 19). The characteristics of study participants are summarized in Table 1.

**Table 1:** Sociodemographic characteristics and diagnoses of the study participants (*N* = 1,327)

Characteristic	*n* (%)
**Age (years)**,** Median (range)** *	53 (18–93)
**Sex**	
Female	1142 (86.1)
Male	185 (13.9)
Race/Ethnicity**	
Brown	612 (46.1)
White	421 (31.7)
Black	293 (22.1)
Asian	1 (0.1)
**Marital status**	
Single	439 (33.1)
Married/consensual union	594 (44.8)
Divorced/separated	153 (11.5)
Widowed	141 (10.6)
**Monthly household income**	
No income	29 (2.2)
Less than one minimum wage	27 (2)
One minimum wage	378 (28.5)
1 to 2 minimum wages	411 (31)
2 to 4 minimum wages	326 (24.6)
5 to 7 minimum wages	54 (4.1)
8 to 10 minimum wages	14 (1.1)
More than 10 minimum wages	1 (0.1)
Prefer not to disclose	87 (6.6)
**Education level**	
Illiterate	20 (1.5)
Primary school incomplete	167 (12.6)
Primary school complete	253 (19.1)
Secondary school incomplete	124 (9.3)
Secondary school complete	484 (36.5)
Tertiary Education incomplete	78 (5.9)
Tertiary Education complete	165 (12.4)
Postgraduate incomplete	4 (0.3)
Postgraduate complete	32 (2.4)
**Diagnosis**	
Rheumatoid Arthritis	464 (35.0)
Systemic Lupus Erythematosus	451 (34.0)
Vasculitis	110 (8.3)
Ankylosing Spondylitis or Other Spondyloarthropathies	85 (6.4)
Psoriatic Arthritis	70 (5.3)
Systemic Sclerosis	70 (5.3)
Sjögren’s Syndrome	49 (3.7)
Juvenile Idiopathic Arthritis	38 (2.9)
Autoimmune Myopathy (Dermatomyositis or Polymyositis)	32 (2.4)
Antiphospholipid Syndrome	16 (1.2)
**Duration of disease (years)**, Median (Range) ***	13.0 (0.0–53.0) ***

* For the variable age data on median (range) is presented

**Self-reported following the standard classification used by the Brazilian Institute of Geography and Statistics (IBGE)

***Calculated as the time from the year of diagnosis to the year of signing the study’s informed consent form

**Fig. 1:**
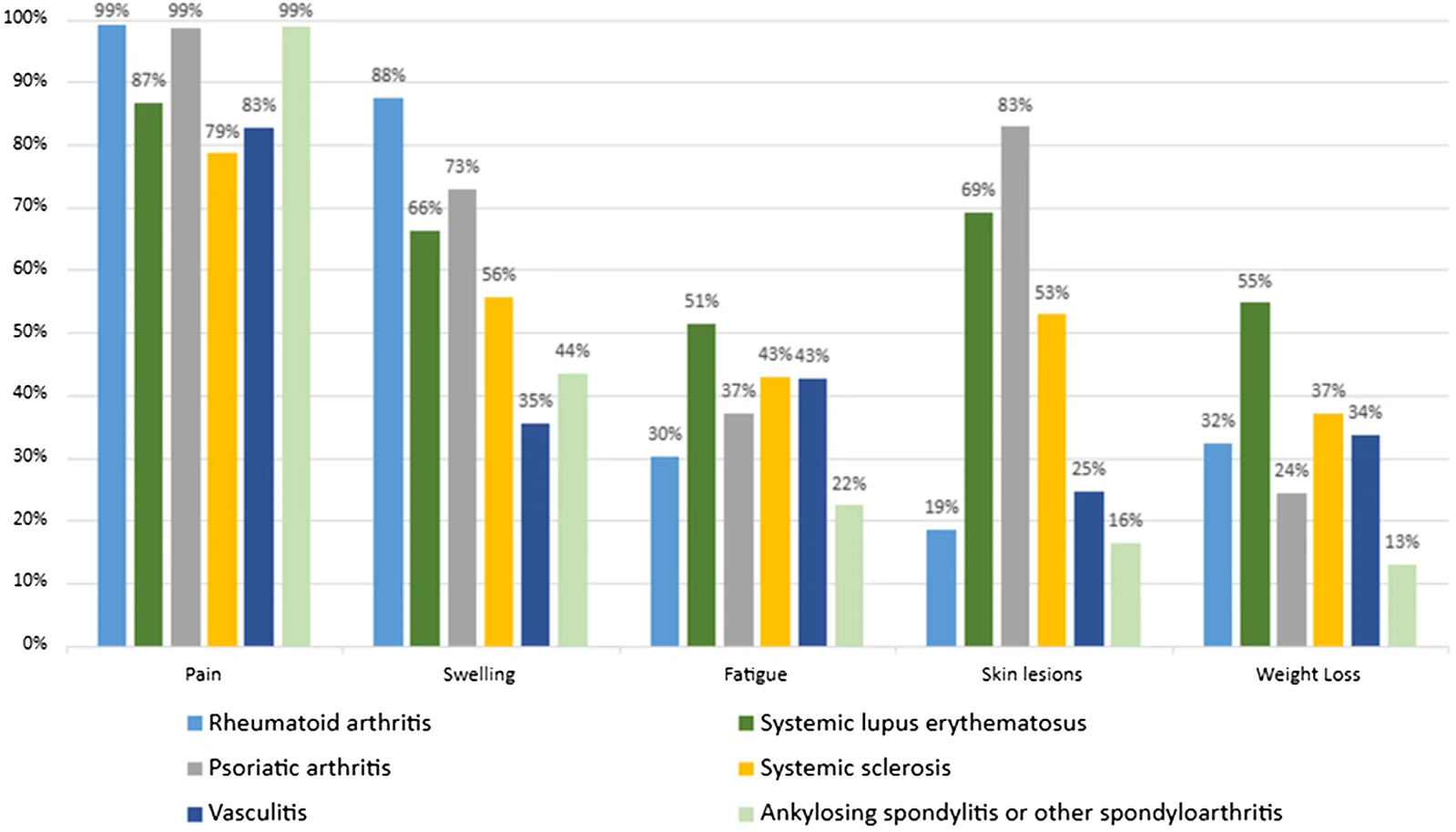
Distribution of the most frequent symptoms according to IMRD

The initial symptoms were diverse, with 91.4% (*n* = 1,213) of the participants reporting pain, followed by swelling (68.5%, *n* = 909), fatigue (39.9%, *n* = 529), and weight loss (38.2%, *n* = 507). A detailed analysis of symptoms according to specific diagnoses is shown in Fig. 1.

Patients sought care at the beginning of symptoms mainly at public emergency units (26%, *n* = 346), primary health centers/family clinics (25%, *n* = 328) and private offices (25%, *n* = 333). The specialties that were initially consulted were non-rheumatologists doctors at public primary care clinics (43.3%, *n* = 574), orthopedists (32.7%, *n* = 434), and emergency physicians (31.9%, *n* = 423). Among the participants, 56.9% (*n* = 755) consulted one medical specialty before reaching a rheumatologist, 28.3% (*n* = 376) consulted two specialties, and 14.8% (*n* = 196) consulted more than two specialties. The diagnosis was confirmed by a rheumatologist in 88.3% of the patients.

Regarding time until the first rheumatologist appointment since the onset of symptoms, the median was 7 months (0,5-216). A stratified analysis was performed to compare the median time for each specific diagnosis against the median time for all other diagnoses combined. The results, shown in Table 2, revealed significant variability in the median time from symptom onset to the first rheumatologist appointment across different diagnoses. PsA and SpA are part of the spondyloarthritis group, and both showed significantly longer wait times, with median times of 20.5 (0.5–192, p-value < 0.001) and 12 months (1.0–200, p-value < 0.001). Conversely, SLE had a significantly shorter median time of 6 months (0,5-216, p-value < 0.001). Other conditions, such as Juvenile Idiopathic Arthritis (JIA, SSc, and SS, did not show significant differences in appointment times, suggesting a more uniform access level, albeit generally reflecting longer wait periods.

**Table 2:** Time since the onset of symptoms to reach the first appointment with the rheumatologist according to diagnosis

Diagnostic	Median time, months (range)	*p*-value^*^
Psoriatic arthritis	20.5 (0.5–192)	< 0.001
Ankylosing spondylitis or others spondylarthritis	12.0 (1-200)	< 0.001
Systemic Sclerosis	12.0 (1-120)	0.142
Sjögren’s Syndrome	12.0 (1-156)	0.262
Vasculitis	12.0 (1-120)	0.394
Juvenile Idiopathic Arthritis	11.0 (0.5–180)	0.429
Autoimmune myopathy (dermatomyositis or polymyositis)	7.0 (1 -120)	0.978
Antiphospholipid antibody syndrome	6.5 (2-120)	0.831
Systemic lupus erythematosus	6.0 (0.5–216)	< 0.001

^*^ Mann‒Whitney test

The analysis of the number of specialties visited also shows that the median time to the first rheumatologist consultation is 12 months for individuals who visit two or more specialties, compared to 5 months for those who consult only one specialty. The median time to diagnosis was 9 months for patients who consulted one specialty before seeing a rheumatologist, 15 months for those who consulted two specialties, and 17.5 months for those who consulted more than two specialties, as shown in Table 3.

**Table 3:** Time to first rheumatologist appointment and definite diagnosis according to the number of specialties consulted

**First rheumatologist appointment**	**Median Time, months (range)**	***p*** -value**
1 specialty	5.0 (0.5–216)	
2 specialties	12.0 (0.5–192)	< 0.001
More than 2 specialties	12.0 (0.5–140)	
**Definite diagnosis**	**Median Time**,** months (range)**	***p*** -value**
1 specialty	9.0 (0.5–216)	
2 specialties	15.0 (0.5–195)	< 0.001
More than 2 specialties	17.5 (1-144)	

^**^ Kruskal Wallis’s test

The median time between symptoms onset and the final IMRD diagnosis was 12 months (0.5–216 months). This time varied according to identified clinical condition, with SLE patients presenting a shorter interval until diagnosis, with a median of eight months (0.5–216), and PsA patients the longest, with a median of 33 months (2-195), with complete data presented in Table 4.

**Table 4:** Time between symptom onset and definite diagnosis by disease

Disease	Median time, months (range)
Psoriatic arthritis	33.0 (2–195)
Ankylosing spondylitis or other spondylarthritis	24.0 (1–204)
Sjogren Syndrome	18.0 (1–156)
Systemic sclerosis	17.5 (1–120)
Vasculitis	12.0 (1–132)
Rheumatoid arthritis	12.0 (0.5–180)
Juvenile Idiopathic Arthritis	12.0 (0.5–180)
Autoimmune myopathy (dermato/polymyositis)	12.0 (2–130)
Antiphospholipid antibody syndrome	12.0 (3–122)
Systemic lupus erythematosus	8.0 (0.5–216)

The median time from symptom onset to the initiation of specific treatment was 12 months (0.5–360 months). Notably, this period was longer for individuals with PsA, who experienced a median delay of 33 months, and shorter for those with SLE, with a median delay of 8 months.

This study examined access to treatment for patients with IMRD. Most participants (90.2%, *n* = 1197) reported that their treatment was provided by the public health system (SUS). Concerning non-drug, multidisciplinary treatments, only 39.3% (*n* = 522) of participants engaged in alternative therapies. Among these, physical activity was the most common (62.5%, *n* = 326), followed by physiotherapy (22.2%, *n* = 116) and psychotherapy (19.9%, *n* = 104).

**Fig. 2:**
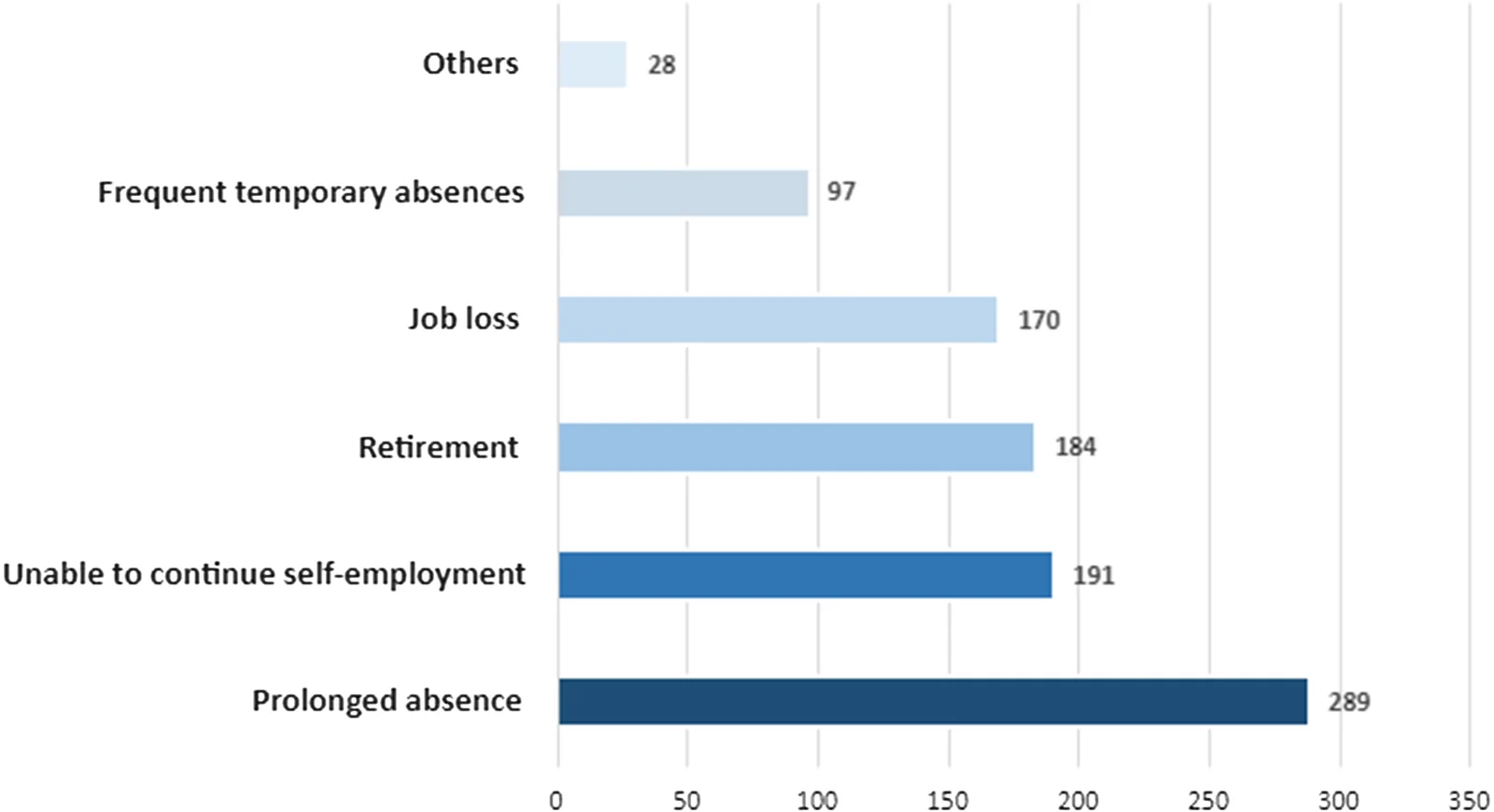
Impact of diagnosis on the participants’ work situation (*N* = 903)

The diagnosis of IMRD negatively affected 85.9% (*n* = 1,139) of the participants, with 48.2% (*n* = 639) noting that it influenced their personal relationships. Additionally, 68.1% (*n* = 903) indicated that their work was affected by the diagnosis, as detailed in Fig. 2, where participants could select multiple responses. Among the diseases, SpA and other spondylarthritis had the most substantial effect on work (*n* = 72, 84%), followed by vasculitis (*n* = 81, 74%), SSc (*n* = 52, 74%), and PsA (*n* = 52, 74%).

In the analysis of emotions reported before and after diagnosis, a statistically significant difference was observed for all emotions except for hope. Throughout the diagnostic journey, negative feelings predominated during the period when the diagnosis was undefined, but there was a noticeable shift towards positive emotions afterward. This shift included increased feelings of happiness, comfort, and well-being, as detailed in Table 5.

**Table 5:** Comparison of feelings before and after the moment of diagnosis

Feelings	Moment of diagnosis		*p*-value^*^
	Before	After	
	No of participants (%)		
***Happy***			< 0.01
No	1305 (98.7)	855 (64.7)	
Yes	17 (1.3)	467 (35.3)	
***Welcomed***			< 0.01
No	1296 (98)	971 (73.4)	
Yes	26 (2)	351 (26.6)	
***Secure/Safe***			< 0.01
No	1307 (98.9)	1201 (90.8)	
Yes	15 (1.1)	121 (9.2)	
***Well***			< 0.01
No	1250 (94.6)	1112 (84.1)	
Yes	72 (5.4)	210 (15.1)	
***Comfortable***			< 0.01
No	1319 (99.8)	1261 (95.4)	
Yes	3 (0.2)	61 (4.6)	
***In suffering***			< 0.01
No	1035 (78.3)	1312 (99.2)	
Yes	287 (21.7)	10 (0.8)	
***Worried***			< 0.01
No	744 (56.3)	1260 (95.3)	
Yes	578 (43.7)	62 (4.7)	
***Lonely***			< 0.01
No	1265 (95.7)	1319 (99.8)	
Yes	57 (4.3)	3 (0.2)	
***Sad***			< 0.01
No	745 (56.4)	1281 (96.9)	
Yes	577 (43.6)	41 (3.1)	
***Hopeful***			0.549
No	1218 (92.1)	1226 (92.7)	
Yes	104 (7.9)	96 (7.3)	

*Comparisons done using McNemar’s test

**Fig. 3:**
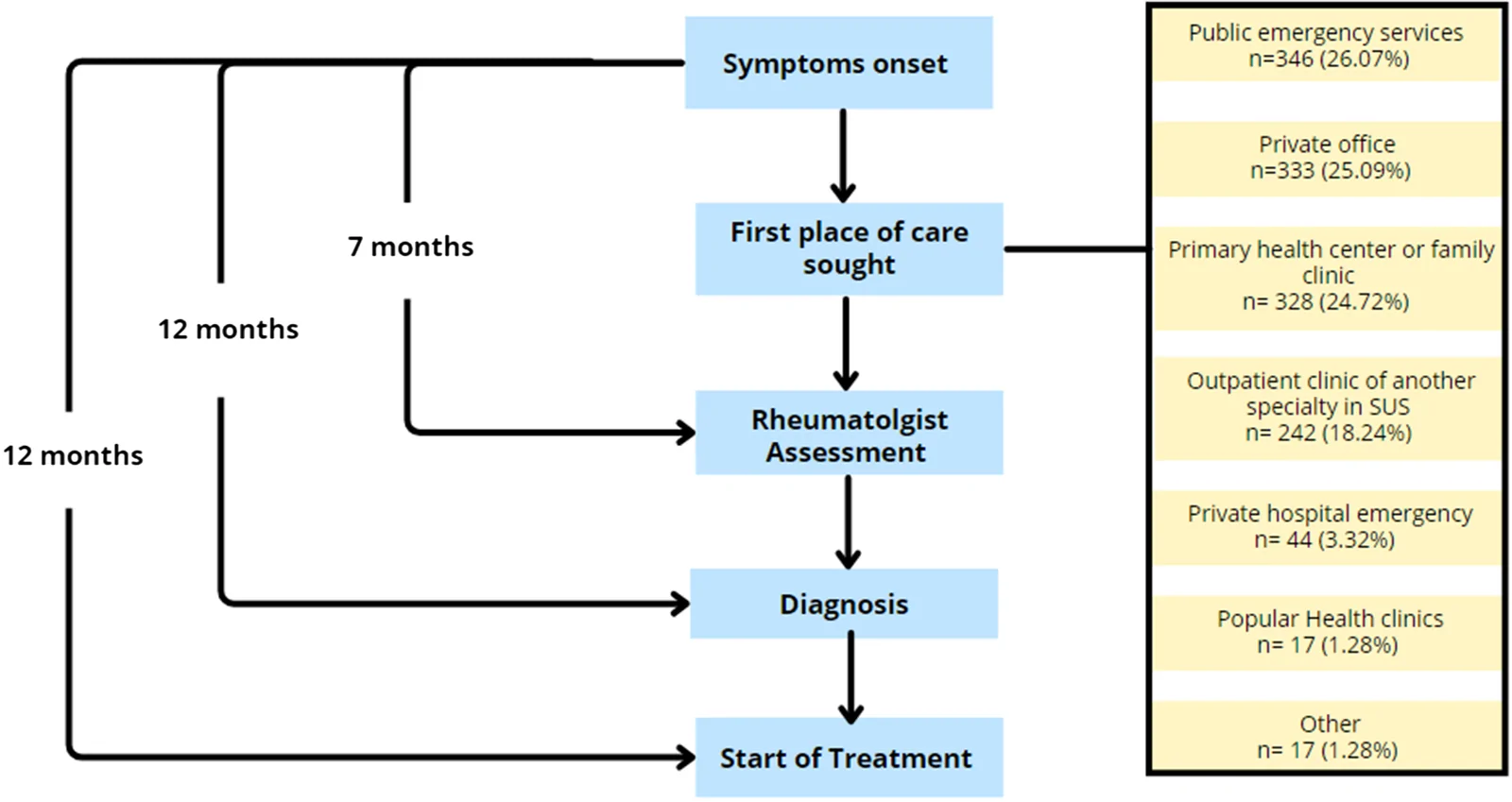
Journey of patients with IMRD, highlighting the median time between the onset of symptoms and consultation with a rheumatologist, diagnosis and start of treatment

The journey of patients with IMRDs is shown in Fig. 3, which summarizes the time from symptom onset to obtaining a rheumatologist assessment, diagnosis and treatment.

## Discussion

This study identified a delay in the healthcare journey of patients with IMRDs, with a median of 12 months from symptom onset to both definitive diagnosis and treatment initiation. This significant delay in recognizing and addressing these conditions can lead to additional complications and negatively impact on patients’ well-being. In addition, the variation in the timing of diagnosis across different clinical conditions highlights the complexity of diagnosis and the need for greater clinical surveillance in certain pathologies, such as PsA and AS [15, 16].

The literature shows that RA patients diagnosed within 3 months of symptom onset have better outcomes in the context known as the “window of opportunity for treatment” [9]. Data from the United Kingdom (UK) and the Netherlands also showed that, most of the time, the delay in referral to a rheumatologist is caused by failures in the primary health care system [17, 18]. A study from the UK analyzed the time between the symptom onset and the first rheumatology consultation in RA patients and found that the median time to see a rheumatologist was 27.2 weeks (6.26 months) [19]. Similarly, a study investigating the time taken for RA patients in eight European countries to be assessed by rheumatologists reported a median of 24 weeks (5.5 months) [20]. Regarding the time from symptom onset to diagnosis, a study in Venezuela found a median of 13 months for diagnosing RA [21], while the REAL study in five regions of Brazil reported a median of 12 months [11]. These findings are consistent with the median time of 12 months found in the present study.

Regarding PsA, several studies highlight the importance of early diagnosis, with delays of more than six to 12 months leading to complications such as peripheral joint erosions and worsening quality of life [22]. Late consultation with a rheumatologist was also identified as a risk factor for unfavorable outcomes. In a study conducted in Spain, a delayed PsA diagnosis of approximately four years from the onset of symptoms was reported [23]. A study conducted in Dublin analyzed 283 patients with PsA and reported a median time of 12 months from disease onset to the first rheumatological assessment [15], similar to the median time of the present study.

For SpA, literature suggests that there is a critical window for initiating treatment, with delays in starting tumor necrosis factor alpha inhibitors (anti-TNF-α) linked to increased radiographic progression [24]. In the UK, the diagnostic delay for SpA has remained stable over time, with a median of 5 years [25]. This delay is likely to be due to SpA often presenting low back pain, causing many patients to initially seek care from non-rheumatologists, who may not immediately recognize the condition. In the present study, the median diagnostic time was 24 months (2 years), making it the second-longest delay among the IMRDs.

PsA and SpA are both part of the spondyloarthritis group and had the longest diagnostic delays in this study. Changes in diagnostic criteria and classification over the past decade have further slowed diagnosis, as updates are not rapidly adopted by non-specialists [26]. Additionally, the need for complex tests like HLA-B27 genetic testing and MRI adds to the challenge. Notably, the genetic test for axial forms of the disease was only incorporated into the SUS in 2024 [27].

This study found that the clinical condition with the shortest time to diagnosis was SLE, with a median of eight months. A cross-sectional study of the LuLa cohort (2021) conducted in Germany, revealed that the median time between the onset of symptoms and the diagnosis of SLE was 13 months. The longer the time until diagnosis, the greater the disease activity, disease-related impairment and fatigue, and the lower the health-related quality of life [16]. Another study carried out by Kpsala et al. (2023) using data from the “Attikon” lupus cohort conducted in Greece reported that the median time to diagnosis after the first symptoms was approximately 24 months [28]. Our findings diverge from the general literature regarding the time to lupus diagnosis, with our study reporting a shorter interval until SLE diagnosis. This discrepancy may be attributed to the specific characteristics of our study population, which included patients from tertiary health centers. These patients are often referred directly to specialists or treated as inpatients due to the severity of their condition, factors that typically expedite the diagnostic process.

Analysis of access to health services revealed a variety of pathways taken by participants, with more than half of the participants initially seeking care in emergency departments and primary care clinics. This highlights the need for awareness of the signs and symptoms of IMRD among health professionals at all levels of care, in order to promote earlier and more targeted intervention. The findings indicate a significant delay in referring patients to rheumatologists after symptoms appear, with many patients consulting multiple physicians before receiving the correct diagnosis and referral. A study conducted in Porto Alegre, which assessed waiting times for rheumatology appointments at a tertiary care center also reported lengthy waiting periods, noting that a quarter of patients waited 10 months or longer for an appointment [14].

Regarding the level of educational attainment observed in the study population, according to data from the Brazilian Institute of Geography and Statistics, based on the Continuous National Household Sample Survey conducted in the first and second quarters of 2025 [29], the population aged 14 years or older in the state of Rio de Janeiro shows relatively high educational attainment. However, the study sample presented a lower educational level compared with the state average. This finding is consistent with the socioeconomic vulnerability observed in the study population and may help explain the greater dependence on the public health system for referral to specialized care.

The present study revealed a significant impact of IMRD on work activity. Work disability is a significant outcome for RA patients of working age. A prospective study found that 25% of patients experienced work disability 6.4 years after disease onset, with this number increasing to 50% at 20.9 years, and most cases of disability occurred later in the disease course [30]. A study evaluating work disability among people with SpA reported that 31% were unable to work, with an additional 15% reporting changes in their professional lives attributable to SpA [31]. Other studies also revealed a negative influence of IMRD on work associated with SLE, SS, SSc, vasculitis, PsA, APS, JIA and autoimmune myopathies [32–39]. A study conducted by Xiang et al. (2020) demonstrated that, in comparison to patients with non-autoimmune rheumatic diseases such as osteoarthritis (OA), patients with inflammatory arthritis (IA) are more likely to report reduced work ability and productivity [40].

A qualitative study conducted with SLE patients in the UK highlighted that misdiagnoses and delays in diagnosis were perceived as detrimental, contributing to feelings of insecurity and a persistent fear of not being believed by healthcare providers, even in future positive medical interactions. Participants also reported a gradual decline in self-confidence over time [41]. In the current study, hope did not exhibit significant changes before and after diagnosis, indicating that participants remained largely low in hope regarding their condition. Conversely, research by Schiavon et al. (2017) emphasized the pivotal role of hope in managing chronic diseases, showcasing its positive impact on coping strategies, treatment adherence, and overall psychological well-being [42].

The present study has certain limitations that should be considered. The cross-sectional nature of the study, which investigates events that occurred many years earlier, poses a risk of memory bias among participants. This lapse in recall could affect the accuracy of the reported information. Additionally, the study’s focus on patients from tertiary hospitals, which serve as referral centers, may limit the generalizability of the findings. These hospitals typically serve a population with access to specialized care, potentially misrepresenting the broader patient population, particularly those without such healthcare access. Consequently, the results may not fully reflect the experiences of patients managed in primary or secondary care settings.

On the other hand, the study’s large sample size strengthens the reliability of its findings. It encompasses a diverse range of immune-mediated rheumatic diseases, offering a comprehensive understanding of various patient challenges and its mixed-methods approach, combining qualitative and quantitative analyses, capturing both statistical trends and the emotional and psychological impacts of delayed diagnosis, providing a holistic view of the patient experience.

## Conclusion

This study provides valuable insights into the multifaceted challenges encountered by patients with IMRDs. It highlights critical issues such as diagnostic delays, barriers to treatment access, and the significant impact these factors have on patients’ well-being, especially in a vulnerable population with socioeconomic fragility, which relies heavily on the public health system. There is a clear need for knowledge of dissemination efforts focused on spondyloarthritis, particularly for primary health care and emergency professionals, given the substantial diagnostic delays and greater impact on work. By incorporating a large and diverse patient sample and utilizing both qualitative and quantitative methodologies, the study underscores the complexity of managing these conditions and the urgent need for comprehensive, multidisciplinary interventions to improve patient care and outcomes.

## Electronic Supplementary Material

Below is the link to the electronic supplementary material.

Supplementary Material.

## Data Availability

The authors confirm that the data supporting the findings of this study are available within the article and its supplementary materials.

## Abbreviations

IMRD: Immune-mediated rheumatic diseases
RA: Rheumatoid arthritis
APS: Antiphospholipid syndrome
SpA: Spondylarthritis
JIA: Juvenile idiopathic arthritis
OA: Osteoarthritis
SLE: Systemic lupus erythematosus
SS: Sjögren's syndrome
DALYs: Disability-adjusted life years
SSc: Systemic sclerosis
SMR: Standard mortality ratio
DMARDs: Disease-modifying antirheumatic drugs
SUS: Brazil's Unified Health System
HUPE: Pedro Ernesto University Hospital
PPC: Piquet Carneiro Polyclinic
UFRJ: Federal University of Rio de Janeiro
HUCFF: Clementino Fraga Filho University Hospital
UK: United Kingdom
anti-TNF: Tumor necrosis factor alpha inhibitors
EAMs: Extra-articular manifestations
