# Supplementary material for: Understanding barriers: patient experience with immune-mediated rheumatic diseases in Brazilian public health
Source: Adv Rheumatol. 2026 Aug 19;66:65. doi: 10.1186/s42358-026-00563-x (PMC13753221; doi:10.1186/s42358-026-00563-x)
Supplement: Supplementary file 1 [file 42358_2026_563_MOESM1_ESM.docx]

# Data Collection Instrument

**DEMOGRAPHIC DATA**

Study Registration Number:

Hospital Medical Record Number:

What is your marital status?

o Single
o Married / stable union
o Divorced / legally separated
o Widowed

What is your level of education?

o Illiterate

o Incomplete elementary education

o Complete elementary education

o Incomplete high school

o Complete high school

o Incomplete higher education

o Complete higher education

o Incomplete postgraduate education

o Complete postgraduate education

o Prefer not to answer

What is your race or ethnicity?

o White / light-skinned

o Brown / mixed / multiracial

o Black

o Indigenous

o Asian

What is your monthly family income?

o No family income
o Less than one minimum wage
o One minimum wage
o 1 to 2 minimum wages
o 2 to 4 minimum wages
o 5 to 7 minimum wages
o 8 to 10 minimum wages
o More than 10 minimum wages
o Unknown
o Prefer not to answer

**Any additional comments?**
o No
o Yes. If yes, please describe:

**CLINICAL ASSESSMENT**

**What is your diagnosis?** (Multiple answers allowed)

➢ Juvenile Idiopathic Arthritis

➢ Rheumatoid Arthritis

➢ Psoriatic Arthritis

➢ Ankylosing Spondylitis or other spondyloarthritis

➢ Systemic Sclerosis

➢ Systemic Lupus Erythematosus

➢ Autoimmune Myopathy (dermatomyositis or polymyositis)

➢ Sjögren’s Syndrome

➢ Antiphospholipid Syndrome

➢ Vasculitis

**What were your first symptoms?**

➢ Pain

➢ Swelling

➢ Fatigue

➢ Fever

➢ Skin lesions

➢ Loss of strength

➢ Weight loss

➢ Hair loss

➢ Oral ulcers

➢ Fingers change color in the cold

➢ Urinary symptoms

➢ Pulmonary symptoms

➢ Neurological symptoms

➢ Gastrointestinal symptoms

➢ Thrombotic event

➢ Dry eyes or mouth

➢ Enlarged lymph nodes

➢ Other. If other, please describe:

**What was the first healthcare facility you sought?**

o Low-cost private clinic
o Private medical office
o Private hospital emergency department
o Public hospital emergency department
o Primary healthcare unit or family health clinic
o Outpatient clinic of another specialty in the public health system (SUS)
o Emergency Care Unit (UPA)
o Other. If other, please describe:

**How long did it take for you to obtain your first appointment with a rheumatologist?**
(Answer in months)

Which medical specialties did you consult before seeing a rheumatologist?

➢ Primary care physician (health unit or family clinic)
➢ Emergency physician
➢ Orthopedist
➢ Occupational physician
➢ Other
➢ I did not consult any other medical specialty

If other, please describe:

**Which physician confirmed your diagnosis?**
o Primary care physician (health unit or family clinic)
o Emergency physician
o Orthopedist
o Public health system (SUS) rheumatologist
o Private practice rheumatologist
o Occupational physician
o Other. If other, please describe:

**How long did it take for your diagnosis to be made from the onset of symptoms? (Answer in months)**

**In which year did you receive your diagnosis?**

**Were you living in the state of Rio de Janeiro when you received your diagnosis?**
o No
o Yes

In which municipality of Rio de Janeiro were you living at the time of diagnosis?

**Were you referred to the rheumatologist through SISREG?**
o No
o Yes
o I do not know

**Did you have difficulty undergoing any complementary tests through the public health system (SUS)?**
o No
o Yes
o Not applicable

If yes, which tests were difficult to access?
➢ Laboratory tests
➢ Imaging exams
➢ PPD
➢ Other. If other, please describe:

**Did your diagnosis affect your personal relationships?**
o No
o Yes

If yes, how did it affect them?
➢ Negatively
➢ Positively

**Did your diagnosis affect your work situation?**
o No
o Yes
o Not applicable

If yes, how did it affect your work?
➢ Job loss
➢ Frequent temporary leave
➢ Prolonged leave
➢ Retirement
➢ Unable to continue working as a self-employed worker
➢ Other

If other, please specify:

**During your journey, did you receive or do you currently receive any INSS (social security) benefit due to your diagnosis?**
o No
o Yes
o Prefer not to answer

**During your journey, did you receive or do you currently receive any INSS (social security) benefit due to your diagnosis?**
o No
o Yes
o Prefer not to answer

Regarding the period from the onset of symptoms to receiving your diagnosis, how would you describe how you felt at that time?

➢ Happy
➢ Welcomed
➢ Safe
➢ Well
➢ Comfortable
➢ In distress
➢ Worried
➢ Lonely
➢ Sad
➢ Hopeful

**Any additional information?**
o No
o Yes. If yes, please describe:

TREATMENT

**How long did it take for you to start disease-specific treatment from the onset of symptoms?** (Answer in months)

**Is your treatment provided by the public health system (SUS)?**
o No
o Yes
o I do not know

**At any point, did you obtain your medications through another means?**
If yes, please describe:

How do you access medications for your rheumatic disease?

➢ State pharmacy (RioFarmes) via LME
➢ Obtained at a primary healthcare unit or family clinic
➢ Purchased at a pharmacy
➢ Provided by a health insurance plan
➢ Provided by the hospital where you receive treatment
➢ Through legal action
➢ Other

- If other, please describe:

Do you use any other therapeutic resources?

- No
- Yes If yes, which ones?
- Nutritional counseling
- Physical therapy
- Psychotherapy
- Physical activity
- Acupuncture
- I do not use these therapies
- Other If other, please describe:

If you use or have used these therapies, how do you access them?

- Public health system (SUS)
- Health insurance plan
- Private practice
- Gym
- Free public or philanthropic service
- Other If other, please describe:

Has your disease ever worsened due to interruption of medication use?

o No
o Yes
o I have not stopped taking medication

If yes, what led you to stop taking the medication?

➢ Difficulty purchasing the medication
➢ Difficulty obtaining medication from the public health system pharmacy
➢ Personal decision
➢ Medical decision
➢ Shared decision
➢ Side effects
➢ Difficulty administering injectable medication
➢ Loss of regular medical follow-up
➢ Other

If other, please describe:

Would you be able to maintain your treatment without the support of pharmaceutical assistance from the public health system (SUS)?

o No
o Yes
o I do not know

Are your medical appointments regular?

- No
- Yes

If yes, how often do they occur?

- Once a month or less
- Every 3 months
- Every 4 or 5 months
- Every 6 months
- Once a year
- OtheriIf other, please describe:

Are you usually seen by the same physician?

o No
o Yes

Is the healthcare facility where you receive treatment close to where you live or work?

o No
o Yes

Do you receive any type of transportation assistance for your treatment?

o No
o Yes

What is your main feeling regarding your journey to obtain treatment after receiving your diagnosis?

➢ Happy
➢ Welcomed
➢ Safe
➢ Well
➢ Comfortable
➢ Relieved
➢ In distress
➢ Worried
➢ Lonely
➢ Sad
➢ Hopeful
➢ Other

If other, please describe:

**Any additional comments?**
o No
o Yes. If yes, please describe:

# PANDEMIC IMPACT

Do you believe the COVID-19 pandemic may have delayed your diagnosis?

o No
o Yes
o Maybe
o Not applicable (diagnosis prior to COVID-19

Were there changes in your routine medical appointments?

➢ Appointment cancellation or rescheduling
➢ Loss of follow-up during the pandemic
➢ Problem resolution through electronic means (email, WhatsApp)
➢ Medical consultation by phone call
➢ Teleconsultation (video)
➢ No

Did it make it difficult to undergo tests requested by your physician?

o No
o Yes
o Not applicable

Did it lead to impairment or interruption of your treatment?

o No
o Yes

Was there difficulty accessing medications prescribed for your treatment?

➢ Difficulty purchasing medication due to lack of prescription
➢ Worsening financial difficulty to purchase medication
➢ Difficulty obtaining medication from the primary healthcare unit
➢ Medication shortage at the pharmacy or healthcare unit
➢ Difficulty obtaining medication via LME
➢ Medication shortage at the state pharmacy (RioFarmes)
➢ No difficulty

Have you been vaccinated against COVID-19?

o No
o Yes

Did you experience difficulty getting vaccinated as part of the priority group for immunosuppressed individuals?

o No
o Yes
o I did not try

If yes, what were your difficulties?

- Obtaining a medical report / proof of disease or treatment
- Understanding at the vaccination unit (vaccination center or primary healthcare unit)
- Fear or hesitation about receiving the vaccine
- OtherIf other, please describe:

Any additional comments?

o No
o Yes. If yes, please describe:
